# The Iterative Model of Ethical Analysis for Large-Scale Implementation Of ICT Solutions

**DOI:** 10.37825/2239-9747.1023

**Published:** 2020-10-01

**Authors:** C Dantas, N Machado, S Ortet, F Leandro, M Burnard, C Grünloh, A Grguric, V Hörmann, L Fiorini, F Cavallo, E Rovini, R Scano, M Pocs

**Affiliations:** 1 Innovation Department, Cáritas Diocesana de Coimbra, Portugal; 2 InnoRenew CoE, Slovenia University of Primorska, Andrej Marušič Institute, Slovenia; 3 eHealth Group, Roessingh Research and Development, Enschede, The Netherlands Biomedical Signals and Systems Group, University of Twente, Enschede, The Netherlands; 4 Ericsson Nikola Tesla d.d., Croatia; 5 AGE Platform Europe, Belgium; 6 The BioRobotics Institute, Scuola Superiore Sant’Anna, Italy. Department of Excellence in Robotics & AI, Scuola Superiore Sant’Anna, Italy; 7 Department of Industrial Engineering, University of Florence, Italy; 8 UNINFO - Associazione di Normazione Informatica, Italy; 9 Stelar Security Technology Law Research, Germany

**Keywords:** Active and Healthy Ageing, ICT solutions, pilots, large-scale implementation, Ethics Model, Covid-19

## Abstract

This manuscript presents a model and the methodology to understand and define the ethical management of the large-scale implementation of ICT solutions for Active and Healthy Ageing. Based on project expertise, including experience from the Pharaon project Horizon 2020, this model includes an understanding of the main ethical challenges and the development of the necessary guidelines, measures, and tools for different stakeholder profiles. This model extends beyond conventional ethical guidelines, providing a methodology to actively discuss ethical and societal challenges within a project based on interactive and iterative dialogue between the entire value-chain of stakeholders. One of the cornerstones in the analysis of challenges is focused attention on policy and societal issues that emerge during a project. Accordingly, the model includes targeted reflections and tools delivered in the context of the recent Covid-19 pandemic. The tools developed in this process are organised in a guide that can be actively used throughout large-scale implementation projects related to ICT solutions.

## I. INTRODUCTION

The effective implementation and deployment of ICT solutions at scale comes with several challenges. The ethical analysis of these challenges usually encompasses differing legislation, scattered regulations, and potentially dissonant interpretations. Although this work is challenging to execute in any case and it often reduces the effectiveness of broad adoption and upscaling, proper ethical analysis is not restricted to legislation only. It must go well beyond compliance with European Union (EU) and Member State laws and regulations on fundamental rights, data protection and product liability directives. It is possible to have full compliance with regulations while not considering, with sufficient depth, the best practices towards the people involved, especially when they are potentially vulnerable individuals. This is frequently an underlying issue and is reflected in low market adoption as well as the incongruity between reimbursement and funding mechanisms and the different national rules. Unfortunately, this often leads to a low adoption rate of innovations, especially those products and services derived from funded projects that cannot achieve a broad implementation in the Member States’ daily health and care systems.

As background, it is important to highlight some transversal issues that already preclude a smooth implementation of ICT in health and care across countries or at-scale:

### Differences between European health and care systems

Every european country has its own healthcare system, with two general mainstream systems:

- National Health System (Beveridge) – a healthcare system funded by taxes. In this system, most of the hospitals and doctors work for the government, although private physicians do exist (e.g. England, Portugal), and
- Social Security System (Bismarck) – healthcare costs are covered by more or less mandatory health insurance, which are paid by employer and employee payroll deductions. Everyone has access to care and hospitals and doctors may not operate for profit. (e.g. Germany).

These differences in national systems and funding policies are a major obstacle to upscaling successfully proven solutions in a straightforward manner as it requires a much more complex organisational model. Consequently, different ethical challenges arise connected to differential access to health and care services and solutions between unemployed or vulnerable people, between urban and rural implementations, and between age groups (especially considering the digital divide present between them).

### Differences in national regulations

European countries have different regulatory approaches in health and care delivery and administration, reimbursement schemes, procurement procedures, data exchange between public and private actors, and medical ethics clearance procedures, amongst other domains. These differences create strong impacts on the broad implementation of ICT solutions and thus on ethical challenges as well.

### Covid-19: challenges and opportunities

It would be impossible to reflect on societal challenges and their ethical implications and not consider the effects of the Covid-19 pandemic. During this pandemic period, many traditional services for the ageing population were appropriately closed or limited (e.g. day-care centres, visitor access to retirement homes). Hospitals were crowded and people were afraid to go to their General Practitioner (GP) or primary care services in person. Loneliness and lack of family support became more pronounced due to isolation and travel restrictions. Digital services, if correctly implemented and accessible, could be an ideal tool to address many of these challenges. In this context, a substantial ethical challenge to address will be the redefinition of the balance between digital tools and human presence. Although this might have been perceived as stable in the public’s opinion, the emergency period polarised opinions once again and a greater sensitivity in approaching the testing and implementation of these solutions in the communities will be required.

These are some of the transversal societal challenges that may impede the successful implementation of digital tools in health and care and need to be addressed in any ethical analysis.

**Fig. 1:**
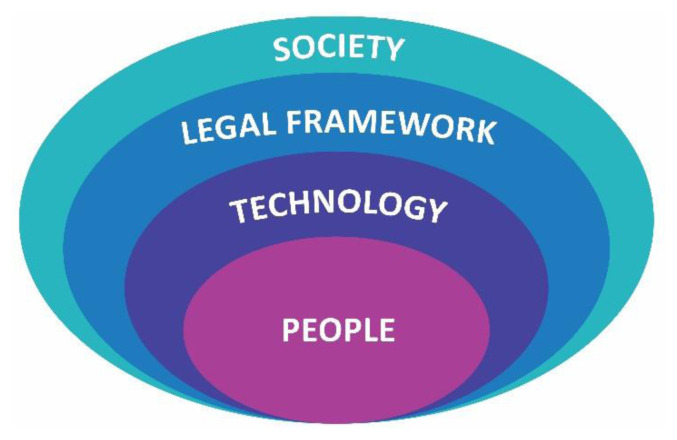
Ethical dimensions

To address these challenges, the first main guiding principle of our Model of Ethical analysis is to enunciate, analyse and discuss the different **Ethical Dimensions**. We identify these dimensions as four sections: **Society, Legal framework, Technology and People** (fig. 1). Within these four sections, several ethical perspectives are presented in sub-sections which were inspired by the scheme of Anunciação [1] [2]. We elaborate on the main sub-sections within each section, explaining how the model will approach them and concluding with a summary of the main goals or associated guidelines to foster.

## II. METHODOLOGY

**Fig. 2:**
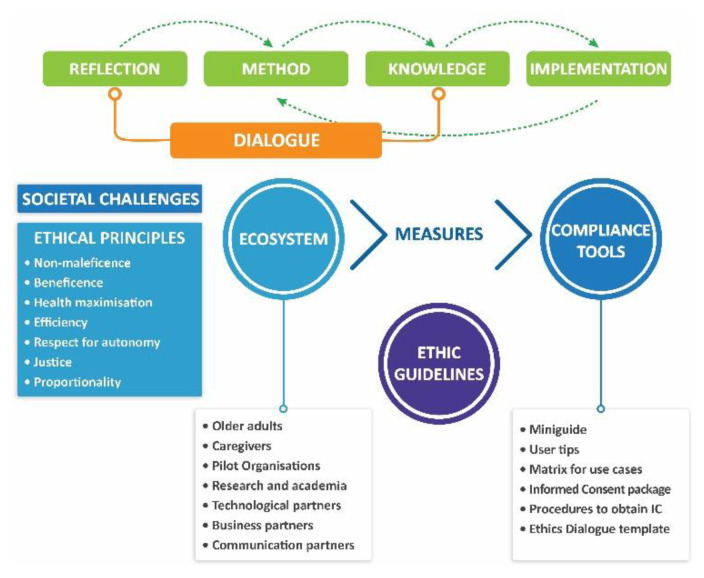
Proposed model for ethical analysis

The Model of Ethical analysis presented in this article intends to achieve better and more efficient results in the large-scale implementation of digital tools for health and care by using a comprehensive and iterative ethics framework that includes a constant dialogue amongst partners, a user-centred co-creation approach and dynamic guidelines for the different stakeholders (fig. 2).

The workflow of ethical analysis is never complete, as new challenges are always emerging. Nonetheless, they can all be addressed if the ecosystem of involved actors develops a broad dialogue, having ethical principles as their first transversal principle alongside objective guidelines and tools developed as their instruments.

For efficiency reasons, a very pragmatic approach was developed, providing a targeted and focused package of guidelines, tools and checkpoints. The approach can be used as a single model or in separate sections that are organised in an Ethics Miniguide and a document of Ethical Guidelines, that include users tips, a matrix for use cases, an informed consent package, procedures to obtain informed consent and the ethics dialogue template.

## III. RESULTS

As previously presented, this Model of Ethical analysis approaches four ethical dimensions: Society, Legal framework, Technology and People.

### Society

Within the societal challenges, three sub-sections are included as transversal**: a) Accessibility, b) Gender, and c) Safety**.

#### a) Accessibility

Different areas of accessibility are relevant: those directly connected to digital accessibility, but also those that refer to housing conditions, mobility, and participation. Making documentation and outputs accessible is key, as well as the accessibility of the technology itself so that it is viable for the user in their own context.

#### b) Gender

Gender best practices include some practical measures, such as gender balance when selecting participants for co-creation activities and validation; evaluation of products and services that include a focus on arising gender issues; ensuring representative data collection to provide adjusted sex/gender specifications; promoting non-sexist language in any documentation generated, especially intervention documents and dissemination materials.

#### c) Safety

Apart from the physical safety of older people in their residences, another important aspect is safety in digital (and online) environments. Awareness-raising on digital security is essential to avoid potential dangers when using the Internet and other technologies, e.g. cyberbullying, phishing, fake news.

### Legal framework

The second ethical dimension is concerned with the study and analysis of the law to guarantee compliance to ethical, privacy and data protection regulations and best practices, thus including hard and soft law. High-level principles, such as respect for the individual as a core component of the Helsinki Declaration should be included [3].

### Technology

The third ethical dimension concerns Technology, a vast topic that encompasses many sub-sections, from which we highlight: **i) Training and education, ii) Building Trustful Artificial Intelligence (AI) with User-Centric Design, iii) Health data silos**, **iv) Data governance, ownership and the FAIR principles, and v) Incidental findings**.

Many discussions are now taking place following the implementation of the EU’s GDPR (General Data Protection Regulation) concerning the question of data ownership. These include if citizens should become custodians of their data or if other data sharing solutions like sharing for research purposes or public services use (e.g secondary data) should be preferred. Another essential dimension is needed in this discussion, that is not concerned with the use of data for research purposes, but with the citizen’s right to access, share and delete their data; the citizen’s will and knowledge to do so; and the system’s tools that can allow an effective interaction. The current lack of a coherent ethical framework on these topics contrasts with other professions such as Medicine or Law, which have codes of ethics and possible penalties in place for noncompliance. Within the ICT field, there are multiple professional bodies based on particular ICT specialities, some of which have their own codes of ethics, but there is no coordinated approach to ethics taken by the ICT industry as a whole. Several related initiatives are developing, such as the European Ethics Framework for the ICT Profession and their results should be considered in future works[4].

#### i) Training and education

Responsible Research and Innovation (RRI) is a growing area, especially in the EU, that draws from classical ethics and aims to provide tools to address ethical concerns from the very outset of a project. When incorporated into a project’s design phase, RRI increases the chances of design being both relevant and strong in terms of ethical alignment.

#### ii) Building Trustful Artificial Intelligence (AI) with User-Centric Design

AI technology should be developed in a way that puts people at its centre and is thus worthy of the public’s trust. This implies that AI applications should not only be consistent with the law but also adhere to ethical principles and ensure that their implementations avoid unintended harm [5]. One example of work in this direction is the field of XAI (Explainable AI) working towards making results of the AI systems understood by humans.

#### iii) Health data silos integration

For healthcare to progress and step beyond the current siloed approach, real-time communication and clear added value for all key stakeholders are the key aspects. A concerted European approach (as the European Health Data Space) and investment in interoperability and essential areas are required to progress [6].

#### iv) Data governance, ownership and the FAIR principles

The question of who owns data, how it is managed, the implications of FAIR (Findable, Accessible, Interoperable, Reusable) data are still not adequately addressed. For example, FAIR data guidelines request permanent and persistent access to data, yet the GDPR declares that data subjects (users) have the right to have their data deleted. The benefits of FAIR data are clear – for validation, educational, and reference. However, potential contradictions between FAIR data, the GDPR, the users’ wishes, and other ethical guidelines must be resolved to protect users and support research, development and innovation.

#### v) Incidental findings

Usually, only the anticipated incidental findings are considered, as defined by the theory of the Bioethics Commission: “Practitioner aims to discover A, but learns B, a result known to be associated with the test or procedure at the time it takes place”[7]. However, in projects that deal with the wellbeing and quality of life, an incidental finding may be the discovery of depression or another psychological condition, which until then was neither known by the person nor previously diagnosed. They must be appropriately analysed to determine whether the incidental findings should be used or not, and how the individual should be informed.

### People

The fourth ethical dimension concerns **People** and is divided into three sub-**sections a) Quality of Life (QoL), b) Older Adults, and c) Workforce**.

#### a) Quality of Life (QoL)

The Organization for Economic Co-operation and Development (OECD) describes 8 points that indicate good quality of life and wellbeing[8]: health status, work-life balance, education and skills, social connections, civic engagement, environmental quality, personal security and subjective wellbeing. This framework is (or should be) taken into consideration as the background of any product or solution developed in the areas of health and care.

#### b) Older adults

According to the Specialist Research Ethics Guidance Paper, in research involving older people, some concerns should be addressed [9]:

##### i) Age

In previous years, users 50+ have been involved in several studies and projects on prevention, aiming to understand what may be needed in the future for ageing populations. However, the situation of a 60 and a 90-year-old may differ substantially regarding physiology, life experience and attitude. Combining wide-ranging age groups into one only category neglects this heterogeneity and does not allow for reliable comparisons.

##### ii) Concepts

The most suitable names to apply to individuals and the collective age groups are much debated. In Europe today, the most widely used and acceptable term for older people is, for an individual, ‘older adult’, and for the collective, ‘older people’, ‘older persons’ or ‘older adults; not elderly or old.

##### iii) Co-creation

Participation is empowerment. Although there is a need to involve multiple stakeholders such as health and care providers and civil society organisations, it is critical to understand that the main stakeholders to be consulted are older adults themselves.

##### iv) Vulnerability

Vulnerability is usually defined by identifying high-risk groups, like the poor, childless, frail, or isolated. However, the vulnerability can and should be seen as the outcome of complex interactions and can only be properly assessed by identifying the contextual conditions, as well as the personal status of each person. Although age can be a vulnerability factor and thus older adults are often considered a vulnerable population, structural ageism is a form of discrimination. Therefore, ageing is not, as such and by itself, considered a vulnerability factor in ethics since it is a process that can be developed in a variety of different ways and is not always associated with particular experiences of vulnerability [10].

##### v) Obtaining Informed Consent

One of the most challenging ethics issues is balancing the need for all of the required documentation for research and ethics clearance purposes (and its jargon) and the adequacy of language that is necessary to obtain proper and freely given informed consent, namely from those who: (a) in some way are dependent on family carers, professional carers or others, (b) live in institutional settings and, most especially, (c) have mild impairments in terms of cognitive capacity.

##### vi) Missing E-Inclusion

The digital divide is a special case of social exclusion. Access, literacy and economic capacity are essential to ensure that inequalities do not drive the development of a new product or service.

#### c) Workforce

Digital health literacy implies connecting literacy skills to functional and critical skills, such as navigating the healthcare system, communicating with healthcare providers, and shared decision making. This demands complementary skills. Health and care professionals need adequate facilities, equipment and consumables, backed by adequate funding, strong health plans and evidence-based policies. They also need knowledge and skills that allow them to maximise the benefits of digital technologies, already in use or the forthcoming. These aspects need to be considered in context analysis.

## IV. DISCUSSION

**Fig. 3:**
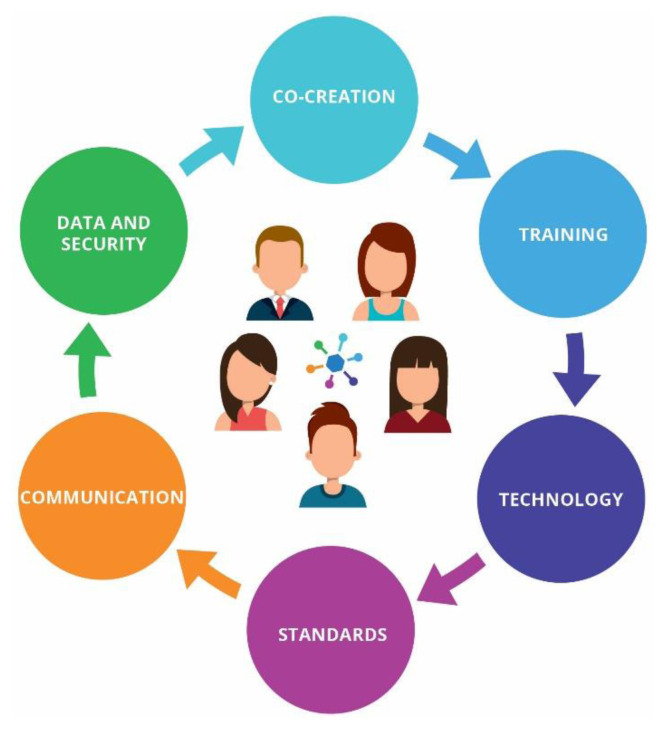
Critical issues for continuous review, analysis and discussion in the model

The challenges and ethical dimensions presented in the previous sections are the ones to be iteratively discussed and analysed through a stakeholder dialogue, in the context of the development of a product, service or project [fig. 3]. The final stage of this model is the use of the Guidelines and Tools Kit that comprises:

### i) Ethics miniguide

A folder that aggregates the areas and works as an interactive index to search and find the guidelines referring to each of the issues addressed.

### ii) Ethical guidelines

A document that provides a clear and focused, yet solid description of each of the specific measures recommended regarding the ethical dimensions previously described, organised in a user-friendly structure to facilitate implementation.

### iii) Users tips

A one-pager that provides useful tips for each of the ethics dimensions for older adults.

### iv) Matrix for use cases

A template with a set of potential questions that may come up and should be discussed between pilot sites, users and providers in order to elicit all the questions; understand if they are all addressed in the use cases; assess which are blocking issues and which are acceptable and to which degree.

### v) Informed consent package

An information packet containing the factsheet, informed Consent (IC) template and revocation form, as well as the guidelines to collect consent in contingency situations [11] [12].

### vi) Procedures to obtain informed consent

A collection of the procedures to be applied, including guidelines to require and gain consent in pandemic situations.

### vii) Ethics dialogue template

Sparks an iterative dialogue and periodic assessment of the best practices applied at the implementation site or between stakeholders, and used at specific checkpoints, or as requested by an oversight body.

## V. CONCLUSION

Some more specificities are likely to arise during the large-scale implementation of the ICT solutions. Therefore, all refinements that may be necessary must be assessed as needed and at regular intervals to assure the model continues to be iterative, agile, pragmatic and valuable.

The toolkit examples of Pharaon are available on the website of the project to be consulted and benchmarked: Full version document [13], Miniguide [14] and Ethical Guidelines [15].
